# Seumetry: a versatile and comprehensive R toolkit to accelerate high-dimensional flow and mass cytometry data analysis

**DOI:** 10.1093/bioinformatics/btag730

**Published:** 2026-10-13

**Authors:** Malte Borggrewe, Markus Flosbach, Lucia Testa, Cedric Ly, Martin Baumdick, Immo Prinz, Hamburg Intestinal Tissue Study Group, Felix J Flomm, Sebastian Schloer, Alaa Akar, Cornelius Flemming, Niklas Jeromin, Johann Rische, Ana Jordan-Paiz, Julia Jäger, Ole Hinrichs, Deborah Sandfort, Jakob Malsy, Konrad Reinshagen, Christian Tomuschat, Nathaniel Melling, Ingo Königs, Elena Billeb, Stefan Bonn, Madeleine J Bunders

**Affiliations:** III. Department of Medicine, University Medical Center Hamburg-Eppendorf, Hamburg, Germany; Department of Virus Immunology, Leibniz Institute of Virology, Hamburg, Germany; Institute of Medical Systems Biology, Center for Biomedical AI (bAIome), Center for Molecular Neurobiology Hamburg (ZMNH), University Medical Center Hamburg Eppendorf, Hamburg, Germany; Hamburg Center for Translational Immunology, University Medical Center Hamburg Eppendorf, Hamburg, Germany; III. Department of Medicine, University Medical Center Hamburg-Eppendorf, Hamburg, Germany; Department of Virus Immunology, Leibniz Institute of Virology, Hamburg, Germany; Hamburg Center for Translational Immunology, University Medical Center Hamburg Eppendorf, Hamburg, Germany; III. Department of Medicine, University Medical Center Hamburg-Eppendorf, Hamburg, Germany; Department of Virus Immunology, Leibniz Institute of Virology, Hamburg, Germany; Institute of Medical Systems Biology, Center for Biomedical AI (bAIome), Center for Molecular Neurobiology Hamburg (ZMNH), University Medical Center Hamburg Eppendorf, Hamburg, Germany; Hamburg Center for Translational Immunology, University Medical Center Hamburg Eppendorf, Hamburg, Germany; Institute of Medical Systems Biology, Center for Biomedical AI (bAIome), Center for Molecular Neurobiology Hamburg (ZMNH), University Medical Center Hamburg Eppendorf, Hamburg, Germany; Hamburg Center for Translational Immunology, University Medical Center Hamburg Eppendorf, Hamburg, Germany; Institute of Systems Immunology, University Medical Center Hamburg-Eppendorf, Hamburg, Germany; III. Department of Medicine, University Medical Center Hamburg-Eppendorf, Hamburg, Germany; Department of Virus Immunology, Leibniz Institute of Virology, Hamburg, Germany; Hamburg Center for Translational Immunology, University Medical Center Hamburg Eppendorf, Hamburg, Germany; Hamburg Center for Translational Immunology, University Medical Center Hamburg Eppendorf, Hamburg, Germany; Institute of Systems Immunology, University Medical Center Hamburg-Eppendorf, Hamburg, Germany; III. Department of Medicine, University Medical Center Hamburg-Eppendorf, Hamburg, Germany; Department of Virus Immunology, Leibniz Institute of Virology, Hamburg, Germany; Hamburg Center for Translational Immunology, University Medical Center Hamburg Eppendorf, Hamburg, Germany; Institute of Medical Systems Biology, Center for Biomedical AI (bAIome), Center for Molecular Neurobiology Hamburg (ZMNH), University Medical Center Hamburg Eppendorf, Hamburg, Germany; Hamburg Center for Translational Immunology, University Medical Center Hamburg Eppendorf, Hamburg, Germany; German Center for Child and Adolescent Health (DZKJ), partner site Hamburg, University Medical Center Hamburg-Eppendorf, Hamburg, Germany; III. Department of Medicine, University Medical Center Hamburg-Eppendorf, Hamburg, Germany; Department of Virus Immunology, Leibniz Institute of Virology, Hamburg, Germany; Hamburg Center for Translational Immunology, University Medical Center Hamburg Eppendorf, Hamburg, Germany; German Center for Child and Adolescent Health (DZKJ), partner site Hamburg, University Medical Center Hamburg-Eppendorf, Hamburg, Germany

## Abstract

**Motivation:**

Recent progress in flow and mass cytometry technologies enables the simultaneous measurement of over 50 parameters for an individual cell. The resulting increase in data volume and complexity present challenges for conventional analysis methods, which are based on manual gating, are time-consuming and fail to capture unknown or minor cell populations. Advances in single-cell RNA sequencing (scRNAseq) technologies have prompted the development of computational analysis tools designed to process and analyze high-dimensional biological data. Building on these advances, we here present Seumetry, a framework that combines flow and mass cytometry data-specific analysis methods with the capabilities of Seurat, a powerful tool for the analysis of scRNAseq data.

**Results:**

Seumetry offers methods for advanced quality control, data visualizations, and differential population abundance and protein expression analysis. We tested Seumetry on an in-house generated complex dataset of immune cells from different layers of human intestines, demonstrating that Seumetry accurately identifies distinct immune cell populations. Furthermore, benchmarking against public datasets showed that Seumetry aligns with manual-gated results, further validating its use for high-dimensional flow and mass cytometry data. In summary, Seumetry provides a new scalable framework for the comprehensive analysis of high-dimensional cytometry data with seamless integration into commonly used scRNAseq analysis tools, enabling in-depth analysis methods to facilitate biological interpretations.

**Availability:**

Seumetry code is available open source under an MIT license and the current version can be viewed and used via Github: https://github.com/imsb-uke/Seumetry; and archived on Zenodo: https://doi.org/10.5281/zenodo.21216681. Flow cytometry data generated for this manuscript is available via Zenodo: https://doi.org/10.5281/zenodo.11935872.

## 1 Introduction

Advances in flow and mass cytometry technologies now enable the simultaneous measurement of over 50 parameters per cell in millions of cells per sample. This high-dimensional data poses new demands to existing analysis techniques. Conventional cytometry analysis based on 2-dimensional manual gating, until now the gold standard for cytometry data analysis, faces challenges in scaling for the application of high-dimensional cytometry data. Issues with conventional gating strategies include inefficiency, subjectivity, and a significant risk of missing unknown or small cell populations. Hence, improved and unbiased computational methods are needed to harness the potential of high-dimensional cytometry data.

To date, several computational tools for cytometry data analysis have been developed, primarily designed for the R programming language, including CyTOF workflow (Nowicka *et al*. 2017), diffcyt (Weber *et al*. 2019), FlowSOM (Van Gassen *et al*. 2015), flowCore (Hahne *et al*. 2009), flowAI (Monaco *et al*. 2016), flowViz (Sarkar *et al*. 2008), CytoTree (Dai *et al*. 2021), CITRUS (Bruggner *et al*. 2014), ImmunoCluster (Opzoomer *et al*. 2021), flowWorkspace (Finak *et al*. 2012), and PeacoQC (Emmaneel *et al*. 2022). These individual workflows address specific aspects of high-dimensional cytometry data analysis, including handling flow cytometry standard (FCS) files, preprocessing, dimensionality reduction, clustering based on self-organizing maps, and differential abundance (DA) and differential expression (DE) analysis. However, interoperability between these packages can be limited, and methods for advanced data analysis such as data integration and trajectory analysis are difficult to integrate in some of the most used packages (Saeys *et al*. 2016, Palit *et al*. 2019, Marsh-Wakefield *et al*. 2021).

Advances have been made in high-dimensional cytometry data analysis methods; however, there have been rapid developments in algorithms for analyzing single-cell RNA sequencing (scRNAseq), which offer a wide range of analysis possibilities. Although scRNAseq and cytometry data differ in certain aspects (counts vs intensities; high vs low number of features), specific methods developed for scRNAseq may be useful and applicable to cytometry data as well. One of the most commonly used R frameworks for scRNAseq data analysis is Seurat (Hao *et al*. 2021), which covers different aspects of data analysis from preprocessing to a range of downstream analyses, including dimensionality reduction and clustering. A notable advantage of scRNAseq tools is their interoperability, since most third-party tools that offer additional analysis options such as batch correction (Korsunsky *et al*. 2019, Hao *et al*. 2021) or trajectory analysis (Trapnell *et al*. 2014, Street *et al*. 2018), integrate seamlessly into the Seurat workflow. However, these scRNAseq analysis algorithms are not readily accessible for the analysis of cytometry data. With these possibilities and challenges in mind, we developed the R toolkit Seumetry, a flow and mass cytometry analysis framework that offers state-of-the-art analysis options for comprehensive cytometry data analysis. Seumetry includes a broad range of cytometry-specific algorithms for data handling, while it seamlessly integrates with Seurat, providing access to the latest scRNAseq analysis options.

## 2 Methods

### 2.1 Acquisition and processing of human intestinal tissue

Intestinal human tissues were collected upon surgical interventions for the treatment of intestinal diseases at the University Medical Center Hamburg-Eppendorf. For an overview of donors please refer to Supplementary Table S1, available as supplementary data at *Bioinformatics* online. Human intestinal tissues were processed and immune cells were isolated as described previously (Schreurs *et al*. 2017, Schreurs *et al*. 2019). After mechanical removal of fat and muscular layers, tissues were incubated with Iscove’s modified Dulbecco’s medium (Thermo Fisher Scientific, 10135083) supplemented with EDTA (5 mM, Promega, V4231), 1,4-dithiothreitol (Carl-Roth GmbH + Co. KG, 6908.1), and 1% fetal bovine serum (Capricorn, FBS-11A) to obtain epithelial immune cells. The cell suspension was filtered through a 70 μm cell strainer and intraepithelial lymphocytes were enriched after epithelial cell layer dissociation using density gradient centrifugation (LSM-A, Capricorn). Lamina propria immune cells were isolated by mincing and incubating in Iscove’s modified Dulbecco’s medium supplemented with 1 mg/mL Collagenase D (Sigma-Aldrich, 11088882001), 1% FBS, and 1000 U/mL DNaseI (StemCell Technologies, 07470). The resulting cell suspension was filtered using a 70 μm cell strainer and immune cells were obtained by density gradient centrifugation using 60% standard isotonic Percoll (Sigma-Aldrich, 17–0891-62).

### 2.2 Flow cytometry

Intestinal cells were stained for 40 markers using a multi-step staining protocol. For a list of all antibodies and steps, see Supplementary Table S3, available as supplementary data at *Bioinformatics* online. Intestinal cells were thawed and incubated in Hank’s balanced salt solution (Sigma, H-6648) supplemented with 0.1 mg/ml DNAse (Sigma, D-4513) and magnesium chloride hexahydrate (Sigma, M-2670). Subsequently, cells were incubated in staining step 1 (Table S3, available as supplementary data at *Bioinformatics* online) with the live dead UV blue dye 10 min at 4°C, followed by incubation in Human TruStain FcX Fc Receptor Blocking Solution (Biolegend, 422302) for 10 min at 4°C in FACS Buffer (PBS supplemented with 1% FBS). After washing with PBS, cells were incubated with antibodies of staining step 2 (Table S3, available as supplementary data at *Bioinformatics* online) in FACS Buffer for 30 min at 37°C. Finally, antibodies of staining step 3 (Table S3, available as supplementary data at *Bioinformatics* online) were added to the cells in FACS Buffer for 30 min at room temperature. Peripheral blood mononuclear cells (PBMCs) were obtained from donors who provided written informed consent from the Hamburg Healthy cohort. PBMCs from two donors were stained for 14 markers (Supplementary Table S3, available as supplementary data at *Bioinformatics* online). PBMCs were thawed and stained the live dead NIR dye and all antibodies for 20 min at RT in PBS. After washing with PBS, cells were fixed with 4% PFA. Both PBMCs and intestinal cells were washed in FACS Buffer and analyzed with a flow cytometer (Cytek Aurora). Before processing with the analysis, raw data was unmixed and cells were pre-gated on single cells (FSC, SSC) live (live dead UV negative) immune (CD45 positive) cells.

### 2.3 Detection of antibody aggregate events

Cells containing antibody aggregates were identified using two Seumetry functions, detect_aggregate_channels() and detect_aggregates(). detect_aggregate_channels() first identifies channel combinations likely to contain aggregate signal: for each channel pair, cells are subset to double-positive events (intensity above pos_threshold, default 1), and the Pearson correlation coefficient is calculated between channels for these events. Channel pairs with fewer than a minimum fraction of double-positive cells (percent_cells, default 0.5%) are excluded to avoid spurious correlation estimates from small samples. Channel pairs with Pearson R above a threshold (threshold, default 0.7) are flagged for the next step. For each flagged channel pair, detect_aggregates() applies a modified RANSAC algorithm (run_modified_ransac()) to fit the aggregate-specific linear relationship between channels. In each of max_iteration (default 10) iterations, a minimal sample of min_to_fit (default 10) events is drawn from the upper half of the dynamic range in both channels and used to fit a linear model; events with a residual below an inlier threshold (default 0.5) are added, and the model is refit. Candidate models are retained only if their slope falls between 0.75 and 1.25, since aggregate signal is expected to be co-detected with high colinearity across channels. Among retained models, the one with the lowest residual standard error is kept. Events with a residual below fit_threshold (default 0.25) relative to this final model are labeled potential aggregates for that channel pair. Each cell’s aggregate score is the number of flagged channel pairs in which it was labeled a potential aggregate. Cells with a score at or above score_threshold (default 2) are classified as antibody aggregates.

## 3 Results

### 3.1 Overview of the Seumetry workflow

Currently available flow and mass cytometry analysis tools frequently focus on specific aspects of data analysis. Seumetry covers additional aspects of cytometry data analysis ranging from (pre)processing to downstream analyses by wrapping existing tools and implementing novel algorithms (Table 1). Among the currently available tools, CATALYST (Nowicka *et al*. 2017) provides the most comprehensive existing pipeline for mass cytometry data, covering normalization, transformation, dimensionality reduction, clustering, and differential analysis within the Bioconductor SingleCellExperiment ecosystem. ImmunoCluster (Opzoomer *et al*. 2021) and CytoTree (Dai *et al*. 2021) similarly bridge cytometry data to single-cell analysis frameworks. ImmunoClust (Sörensen *et al*. 2015) offers a self-contained, model-based clustering pipeline with automated arcsinh cofactor estimation via variance stabilization. Seumetry differs from these tools in three ways with several advantages. First, rather than operating as a fixed pipeline, it represents cytometry data natively as a Seurat object. This modular design gives users direct access to the full, continuously maintained Seurat ecosystem, including dimensionality reduction, clustering, and the broad range of third-party methods built on the Seurat class. Second, it provides a novel antibody aggregate detection algorithm. Third, it unifies flow and mass cytometry preprocessing with these downstream analyses within a single object and workflow.

**Table 1:** Comparison of Seumetry to other R packages for cytometry data analysis.

	Task	Seumetry	FlowCore	**CyTOF workflow** *	CATALYST	diffcyt	CITRUS	FlowViz	Flow Workspace	CytoTree	FlowSOM	FlowAI	ImmunoCluster	ImmunoClust	PeacoQC
**Preprocessing**	Loading FCS files	X^a^	X	X**	X**	X**	X	X**	X	X	X	X	X	X**	X**
	Compensation	X^a^	X	X	X			X**		X	X	X**	X		X**
	Transformation	X^b^	X	X	X		X	X**	X	X	X	X**	X	X	X**
	Gating		X					X	X						
**QC**	Outlier detection	X										X	X		X
	Aggregate detection	X													
**Downstream analysis**	Access to scRNAseq	X													
	tools via Seurat														
	Dimensionality reduction	X^c^		X	X					X	X		X		
	Clustering	X^c^		X	X		X			X	X		X	X	
	Differential abundance	X^d^		X		X	X				X				
	Differential expression	X^d^		X		X									
**Visualization**	Cytometry specific plots	X	X	X	X			X	X	X			X		
	Sample-level PCA/MDS	X		X	X								X		
	Violin plots	X^c^								X			X		
	Dot plots/Heatmaps	X^c^		X	X					X			X		
	Feature plots	X^c^		X	X					X			X		
	Frequency plots	X		X	X								X		
	Export of FCS files	X^a^		X**	X**										

* CyTOF workflow in combination with CATALYST and diffcyt.

** Only available via flowCore.

a Via flowCore.

b Via flowWorkspace for biexp transformation.

c Via Seurat.

d Via edgeR/limma, FCS, flow cytometry standard, References: CyTOF workflow/CATALYST (Nowicka *et al*. 2017), diffcyt (Weber *et al*. 2019), FlowSOM (Van Gassen *et al*. 2015), flowCore (Hahne *et al*. 2009), flowAI (Monaco *et al*. 2016), flowViz (Sarkar *et al*. 2008), CytoTree (Dai *et al*. 2021), CITRUS (Bruggner *et al*. 2014), ImmunoCluster (Opzoomer *et al*. 2021), flowWorkspace (Finak *et al*. 2012), immunoClust (Sörensen *et al*. 2015), PeacoQC (Emmaneel *et al*. 2022).

To demonstrate the functionality and workflow of Seumetry, we generated a high-dimensional spectral flow cytometry dataset comprising 39 protein markers of human intestinal immune cells (Tables S1 and S2, available as supplementary data at *Bioinformatics* online). The test dataset consists of immune cells isolated from 7 adult human intestines, separated in epithelium and lamina propria layers (Fig. 1). The data was manually pre-gated on single (FSC, SSC), viable (viability dye), immune (CD45 positive) cells and downsampled to a maximum of 20 000 cells per sample, due to varying numbers of cells per sample. This dataset is used to present all functionalities of Seumetry with the aim of annotating intestinal immune cell populations. Of note, Seumetry does not offer gating functionality, thus pre-gating on single and live cells, or cell populations of interest, is recommended.

**Figure 1:**
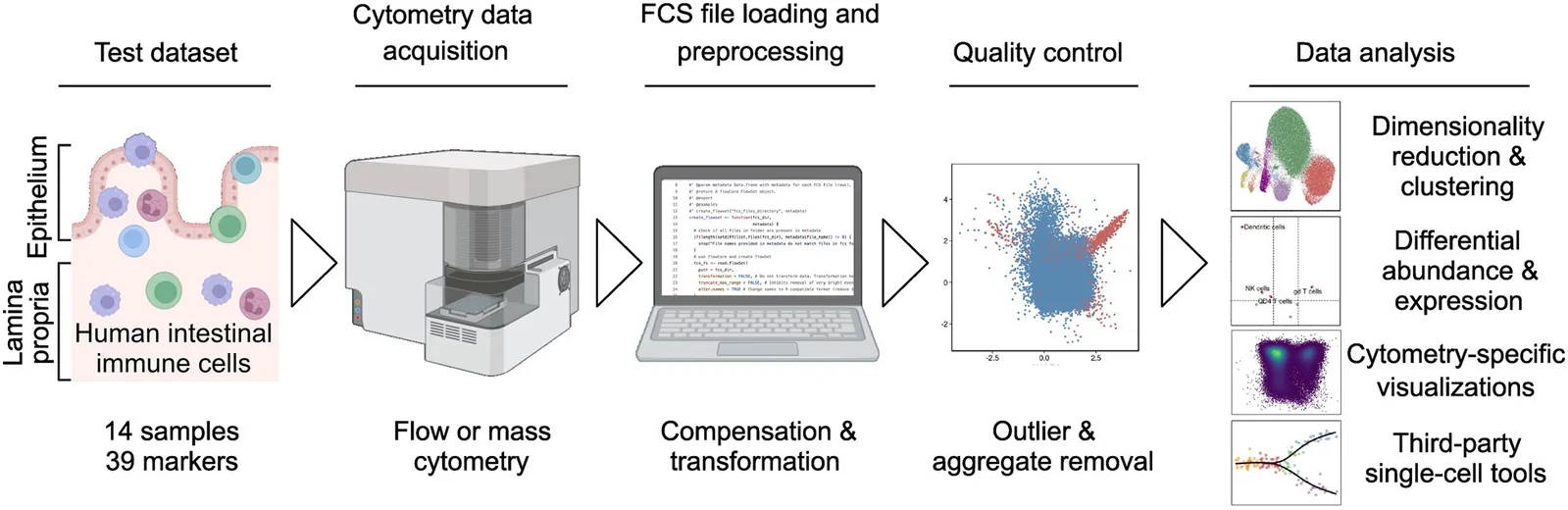
Generation of flow cytometry test dataset and Seumetry workflow. To showcase the Seumetry workflow, we generated spectral flow cytometry data of immune cells in intestinal mucosal samples from 7 adults, including separate analysis of epithelial layer and lamina propria-derived cells. Cytometry raw data is loaded into R and preprocessed, including compensation of protein channels and data transformation. Quality control methods include the detection and removal of outlier events and antibody aggregates. Data analysis options include Seurat data analysis tools, as well as, cytometry-specific clustering, differential abundance, differential expression analysis, additional visualization methods, and potential application of third-party single-cell tools.

After flow cytometry standard (FCS) file loading (create_flowset(); via flowCore package), data is preprocessed in Seumetry including compensation (compensate_data(); via flowCore package) and transformation (transform_data(); biexp via flowWorkspace package) (Fig. 1). Since cytometry data can be inherently noisy, we implemented multiple algorithms to improve data quality, including the identification and removal of outliers (detect_outliers(), remove_outliers_manual()) and antibody aggregate artifacts (detect_aggregate_channels(), detect_aggregates()). Antibody aggregates can occur in highly complex cytometry panels which include a large number of antibodies that can form aggregates and lead to false positive signals (Rasmussen *et al*. 2021). Seumetry contains a novel algorithm for the automated and unbiased removal of these antibody aggregates.

Finally, the high-quality data can be analyzed using common scRNAseq algorithms from other libraries via integration into the Seurat workflow (create_seurat()) including dimensionality reduction, clustering, batch correction, and trajectory analyses (Trapnell *et al*. 2014, Street *et al*. 2018, Korsunsky *et al*. 2019, Hao *et al*. 2021). To expand analysis options, we also implemented additional cytometry-specific clustering (run_FlowSOM(); via FlowSOM Package) and visualizations (plot_pca(), plot_frequency(), plot_cellnumber(), plot_cyto()), and methods for DA (differential_abundance(); via edgeR package) and DE analysis (DE_pseudobulk(); via limma package) (Fig. 1).

We used Seumetry to analyse the in-house generated flow cytometry dataset of intestinal immune cells and publicly available mass cytometry datasets. Based on these analyses we demonstrate that Seumetry can be used for comprehensive analysis of flow and mass cytometry data and accelerate biological interpretation of complex cytometry data.

### 3.2 Data preprocessing and removal of outliers

Seumetry incorporates all necessary flow and mass cytometry-specific data preprocessing and quality control steps by integrating existing functions of other packages and offering new processing algorithms. For cytometry data, compensation may be necessary to correct for spillover effects of protein channels, which is included in Seumetry (compensate_data()) using the compensation matrices embedded in the FCS files or external matrices (Fig. S1A, available as supplementary data at *Bioinformatics* online). The in-house test dataset is a spectral flow cytometry dataset, which also requires unmixing of signals. Seumetry does not offer a functionality for unmixing, as this is usually performed on the acquisition machine and locally calibrated. Next, Seumetry offers cytometry-specific options for data normalization and transformation to stabilize variance and compress high-intensity events. Transformations include biexponential (transform_biexp()) for flow cytometry and the more commonly used inverse hyperbolic sine function (arcsinh) (transform_arcsinh()) for flow and mass cytometry (Fig. S1B, available as supplementary data at *Bioinformatics* online).

When selecting a suitable co-factor for arcsinh transformation, cofactors should be set per marker rather than left at the default (cofactor = 5), and it is advisable to assess visually that transformation resulted in good separation of positive and negative events. Automatic co-factor determination via variance stabilization (e.g. as implemented in the immunoClust package (Sörensen *et al*. 2015)) can also be used. Furthermore, derandomization and bead normalization for mass cytometry data can be included in Seumetry based analyses (bead_norm(); via CATALYST package).

To eliminate undesired outliers in the data, which commonly manifest as very low or high-intensity events in flow and mass cytometry data, e.g. resulting from cell debris, dead cells, or cell clumps, Seumetry offers both manual and automatic workflows. In the manual workflow (remove_outliers_manual()), users can visually inspect each parameter and provide positive and negative thresholds for each channel to remove events. The automatic workflow employs an isolation forest (detect_outliers()), which calculates an outlier score for each event based on all provided antigen channels. Events are regarded as outliers if they surpass a specified outlier score threshold (default: 0.7), based on empirical calibration across representative datasets (threshold may require adjustment for other datasets). Events with an outlier score higher than the threshold can subsequently be removed to obtain high-quality data.

To test the outlier removal workflow on data with genuine low-quality events, we assessed ungated data rather than the pre-gated dataset used elsewhere in this study. Pre-gating on live, single cells already removes most of the dead cells, debris, and clumps the algorithm is designed to detect, so a pre-gated dataset would not provide a meaningful test. We used our generated intestinal immune cells dataset for this validation (*n* = 3; downsampled to 500 000 cells per sample). Although the isolation forest only used measurements of antigen channels, our automatic workflow almost exclusively detected events with unexpectedly high measurements for cell size and granularity (cell clumps), or dead cells (Fig. 2A), indicating that the algorithm removes noisy events while keeping high-quality cells. Subsequently, the algorithm was used to improve the quality of the pre-gated intestinal immune cell test dataset, which resulted in the removal of 60 events with very high or low protein signal intensity (Fig. 2B). Together, this automatic outlier removal method is computationally efficient and can detect noisy events while retaining high-quality cells.

**Figure 2:**
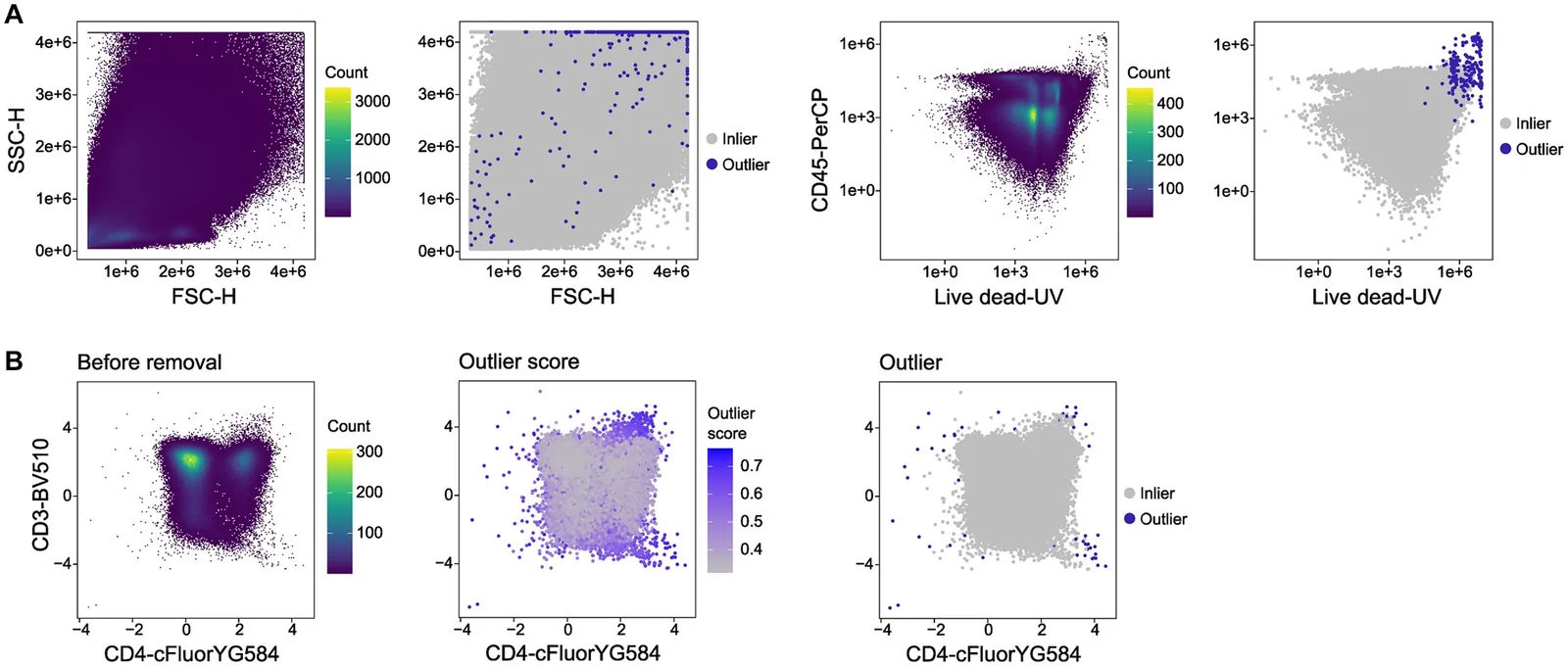
Detection and removal of outlier events in flow cytometry data. (A) Representative two-dimensional density plots and scatter plots illustrating outliers (blue) and inliers (gray) of ungated events showing SSC-H vs FSC-H and CD45-PerCP vs live dead (*n* = 3). (B) Representative plots of outlier detection and removal in test data using CD3 and CD4 as examples (*n* = 14).

### 3.3 Detection and removal of cells with antibody aggregates

The use of large antibody panels in high-dimensional cytometry can introduce additional artifacts due to the aggregation of two or more antibodies, leading to false positive signals on cells (Fig. 1: Quality control). Adjusting laboratory protocols to individually stain cells for each antibody can mitigate these artifacts. However, this approach can be highly time-consuming for large antibody panels, posing new challenges affecting cell viability, staining stability, scaling and feasibility. Alternatively, antibody aggregates can be removed using manual gating, which is time-consuming and subjective, introducing inter-observer variability. To provide an unbiased approach for removing antibody aggregates, we developed a novel algorithm to detect aggregate signals on cells.

In the initial step, the algorithm identifies antigen channels that potentially contain signals of antibody aggregates. Signals of antibody aggregates are expected to be highly collinear double-positive events in specific antigen channel combinations. Thus, the data is subset to double-positive events, which are by default defined as events with an intensity of higher than 1, and a Pearson correlation is performed (detect_aggregate_channels()) (Fig. 3A). A high Pearson R value may suggest the presence of highly collinear events, indicative of cells with antibody aggregates. Applying a threshold for Pearson R (default: 0.7) identifies antigen channel combinations with potential aggregates (Fig. 3B), which resulted in 16 channel combinations with a high likelihood of antibody aggregates in our dataset (Fig. 3C and Fig. S2, available as supplementary data at *Bioinformatics* online). This threshold is deliberately permissive as this step is to detect potential aggregate channels for further inspection. The actual identification of aggregates is performed with the next step.

**Figure 3:**
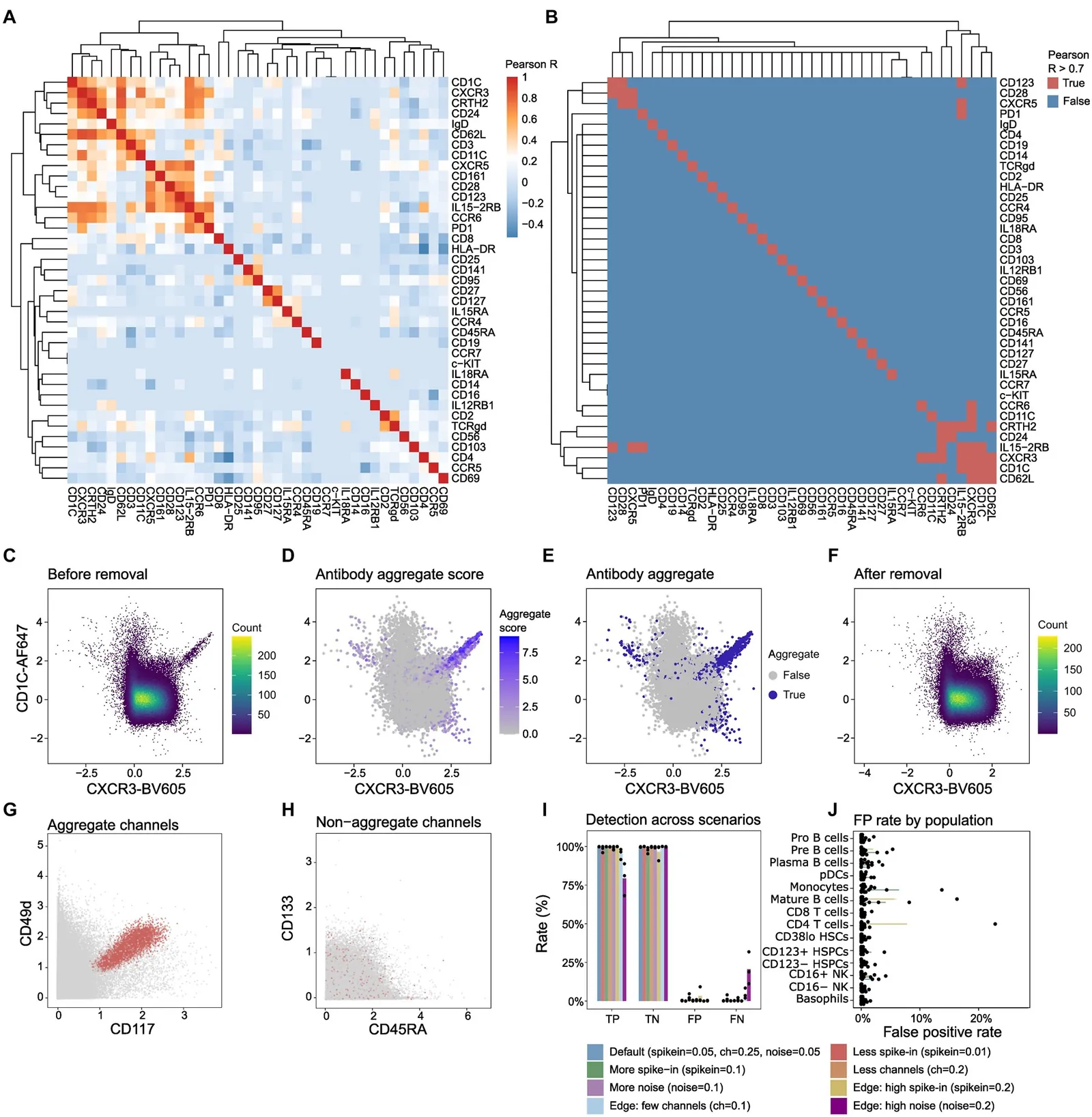
Detection and removal of antibody aggregates. (A and B) Correlation heatmap depicting (A) Pearson R and (B) Pearson R > 0.7 for double-positive events of each channel combination. (C–F) Representative plots of antibody aggregate detection and removal using CD1C and CXCR3 as examples. (C) Two-dimensional density plot of events before removal of antibody aggregate. (D) Scatterplot visualizing aggregate score for each event. (E) Scatterplot depicting antibody aggregates (blue) and non-aggregates (gray) based on an antibody aggregate score threshold of 2. (F) Two-dimensional density plot of events after removal of antibody aggregates (*n* = 14). (G–J) Validation of antibody aggregate detection using simulated aggregate spike-ins on an expert-gated mass cytometry dataset (Levine *et al*. 2015). (G) Simulated antibody aggregate events (red) among background cells (gray) in a representative aggregate channel combination (CD117, CD49d). (H) The same simulated events (red) shown in a non-aggregate channel combination (CD45RA, CD133), where the collinear relationship is absent. (I) True positive (TP), true negative (TN), false positive (FP), and false negative (FN) rates across eight simulated scenarios varying spike-in fraction, fraction of affected channels, and noise level. (J) False positive rate per cell population, pooled across all simulated scenarios.

To identify potential antibody aggregates in the above detected channel combinations, we used a random sample consensus (RANSAC) algorithm, which we modified (run_modified_ransac()). RANSAC was originally developed to fit geometric models to data contaminated with an unknown fraction of outliers, by iteratively sampling minimal subsets of points, fitting a candidate model, and retaining the model supported by the largest set of inliers (Fischler and Bolles 1981). Antibody aggregates produce a similar structure as aggregate-bound conjugates from two channels are physically linked and co-detected roughly in proportion to aggregate size. Therefore, aggregate events should fall along an approximately linear relationship between channels. Genuine double-positive cells, in contrast, arise from independent co-expression of both markers and are not constrained to any fixed ratio, so they scatter more broadly instead of following a single line. We modified the classical algorithm by sampling from the upper 50% of positive events and constraining candidate models to a slope between 0.75 and 1.25, to model only highly collinear antibody aggregate events. Events with a low residual value (default residual value: less than 0.25) based on the fitted linear model are labeled as potential antibody aggregates. The antibody aggregate score indicates the number of channel combinations in which an event is labeled as a potential aggregate (Fig. 3D). To prevent the removal of non-aggregates, events are only considered antibody aggregates if they are labeled as potential aggregates in two or more channels by default (detect_aggregates()) (Fig. 3E and Fig. S2, available as supplementary data at *Bioinformatics* online), and these events are subsequently removed (Fig. 3F). Detection of outliers based on isolation forest (see above) can additionally be performed after removal of aggregates to filter out remaining outliers.

To further validate the antibody aggregate detection algorithm, we performed a spike-in simulation. Synthetic aggregate events were introduced into an expert-gated mass cytometry dataset (Levine *et al*. 2015) and detection performance across a range of scenarios was evaluated (Fig. 3G–J). At default parameters, the algorithm correctly classified nearly all events, with true positive and true negative rates close to 100% and a low false positive rate. False positives were not concentrated in any single cell population (Fig. 3I,J), so genuine double-positive biological populations are not disproportionately misclassified as aggregates. To test performance beyond conditions expected in real biological datasets, we also simulated edge case scenarios with high noise, few affected channels, and high spike-in fractions. True positives were reduced in edge cases, particularly with unrealistically high noise (0.2), which was associated with an increase in false negatives. Importantly, even under edge conditions, the false positive rate did only increase to a maximum average of ∼3% with high spike-in (Fig. 3I).

In summary, the modified RANSAC algorithm for automatic antibody aggregate detection offers an efficient and unbiased approach to removing antibody aggregates, generating improved quality in cytometry datasets.

### 3.4 Dimensionality reduction and clustering of intestinal immune cell populations

A large number of dimensionality reduction and clustering algorithms have been applied to cytometry data, which were compared extensively by others (Weber and Robinson 2016, Wang *et al*. 2023). Dimensionality reduction techniques used are mostly general-purpose/single-cell methods such as PCA, tSNE, or UMAP, whereas clustering algorithms specifically designed for cytometry data have emerged (flowSOM, immunoCluster, etc.). Many general-purpose and single-cell dimensionality and clustering methods are not easily accessible for high-dimensional cytometry analysis via published analysis workflows (Palit *et al*. 2019). These tools include common algorithms for dimensionality reduction (PCA, tSNE, UMAP) and clustering (Louvain, Leiden) and more specialized algorithms such as trajectory analyses and PCA-based batch correction (Trapnell *et al*. 2014, Street *et al*. 2018, Korsunsky *et al*. 2019, Hao *et al*. 2021). Seumetry is a pipeline for researchers to now employ a multitude of scRNAseq algorithms for the in-depth analysis of the flow and mass cytometry data. To expand the tool box with commonly used cytometry clustering algorithms, FlowSOM (Van Gassen *et al*. 2015) was integrated (run_FlowSOM()). Furthermore, Seumetry allows integration of other cytometry-specific third-party tools, for example, for batch correction methods such as CytofRUV, CytoNorm, and cyCombine (Van Gassen *et al*. 2020, Pedersen *et al*. 2022).

To explore the variance between samples, for example, to assess the quality of replicates and differences between conditions, we added a principal component analysis (PCA) based on median protein expression per sample (Fig. 4A). As our dataset consisted of human samples that exhibited considerable donor variation (Fig. 4A), the data was integrated using an anchor-based CCA integration supplied in the Seurat framework (Hao *et al*. 2021). Anchor-based CCA integration and other integration methods such as Harmony (Korsunsky *et al*. 2019) work by correcting PCs, which can be applied to a variety of data sources, where PCs are used for dimensionality reduction and clustering. Here, CCA integration reduced the variance in the PCs based on donor identity (patient_id) considerably, particularly in the first 15 PCs, while variance explained by different mucosal layers was low, but mostly maintained (Fig. S3A, available as supplementary data at *Bioinformatics* online). Subsequently, a uniform manifold approximation and projection (UMAP) and Louvain clusters were computed based on corrected PCs using Seurat (Fig. S3B, available as supplementary data at *Bioinformatics* online). Louvain clustering builds a k-nearest-neighbor graph from continuous coordinates, whether derived from PCA components or marker features directly. Seumetry supports both, so the method works well on continuous cytometry data even though it originated in the scRNAseq field. To identify specific intestinal immune cell populations, differential cluster markers were calculated using a Wilcoxon test (Seurat function “FindAllMarkers”) (Fig. S3C, available as supplementary data at *Bioinformatics* online), and clusters were merged and annotated (Fig. 4B). Although variation in frequencies of cell types across specific donors was still visible (donors 5 and 6, which had rarer diagnoses compared to the other donors), all cell types were present in all donors and frequencies were similar among the other donors (Fig. 4C), demonstrating a successful donor integration. This analysis workflow resulted in the identification of the expected major intestinal immune cell populations with the detection of their known markers (Fig. 4D).

**Figure 4:**
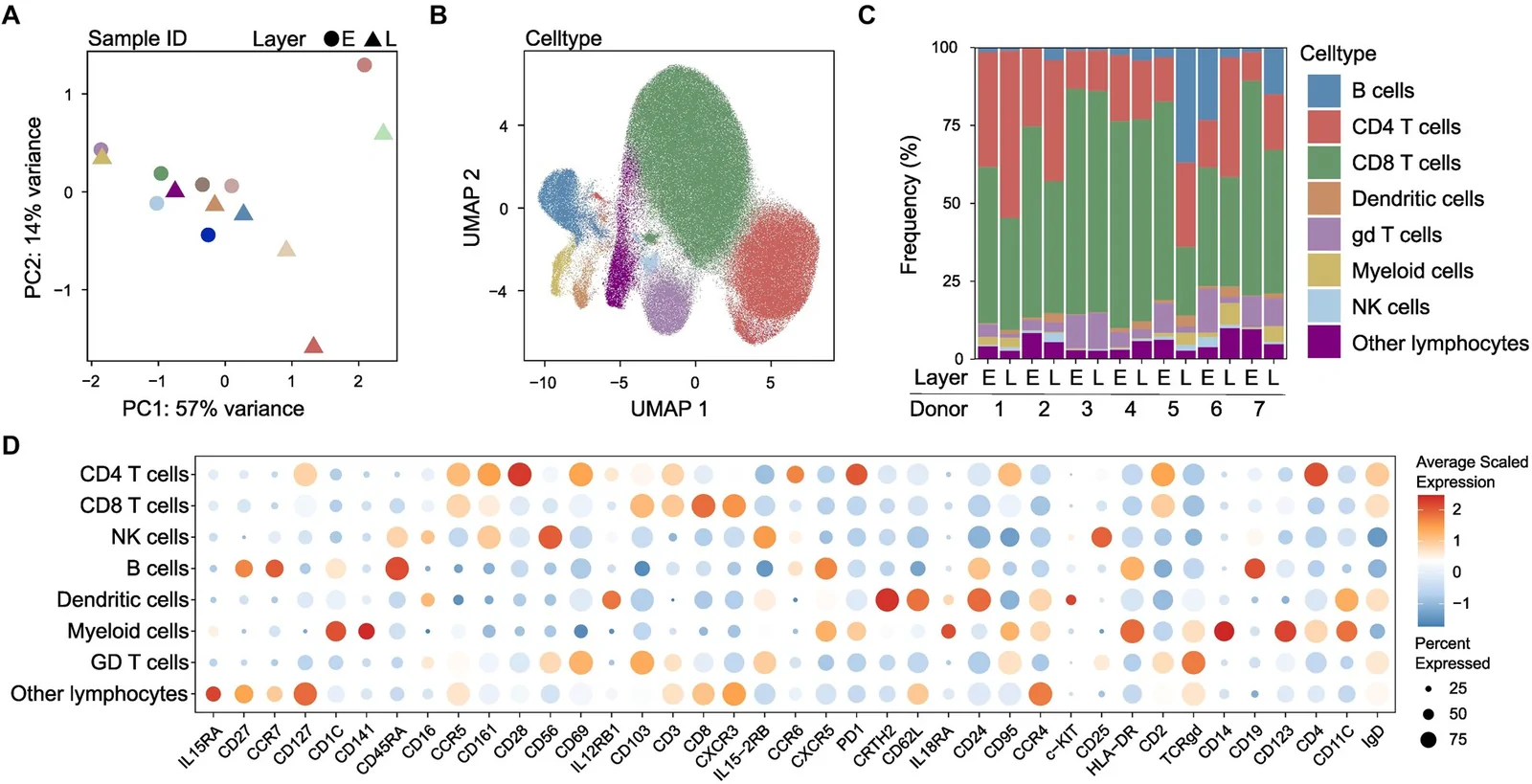
Dimensionality reduction and clustering of intestinal immune cells. (A) Principal component analysis based on median surface protein expression per sample, colored by sample id and shape indicates layer. (B) UMAP of all samples colored by immune cell populations identified by merging and annotating louvain clusters. (C) Frequency plot of immune cell populations across all samples. (D) Average expression (scaled) of all proteins across immune cell populations visualized in a dotplot (*n* = 14). E, Epithelium; L, Lamina propria.

### 3.5 Differential population abundance and protein expression of intestinal immune cells in epithelium and lamina propria

High-dimensional cytometry is commonly used to identify the differential abundance of cell populations or differential expression of proteins across conditions. Hence, we added a DA and DE analysis.

DA analysis in Seumetry utilizes cell numbers per sample and condition to construct a generalized linear model (GLM) using the edgeR R package (Robinson *et al*. 2010). To account for the compositional nature of cytometry cell-count data, where a true increase in one population necessarily reduces the relative frequency of others, cell counts can be normalized using the trimmed mean of M-values (TMM) method supplied by edgeR prior to model fitting. Alternatively, samples acquired with matched input cell numbers can be normalized using this pre-cleanup count instead (before debris, dead cell, or aggregate removal). Pairwise comparisons can be performed, which uses a likelihood ratio test and provides corrected *P* values using the Benjamini-Hochberg method. To demonstrate the DA analysis, we compared the abundance of immune cells between the epithelium and lamina propria layers (Fig. 5A and B). Dendritic cells, NK cells, and CD4 T cells were enriched in the lamina propria, whereas γδ T cells were enriched in the epithelial layer of the intestines. These findings confirm previous findings by our group and others (Mowat and Agace 2014, Sagebiel *et al*. 2019, Schreurs *et al*. 2019). In line with these studies, CD8 T cells were higher in the epithelium (Fig. 5B), albeit not significant (adjusted *P* = 0.15), further validating that Seumetry accurately identifies immune cell populations in an unbiased manner.

**Figure 5:**
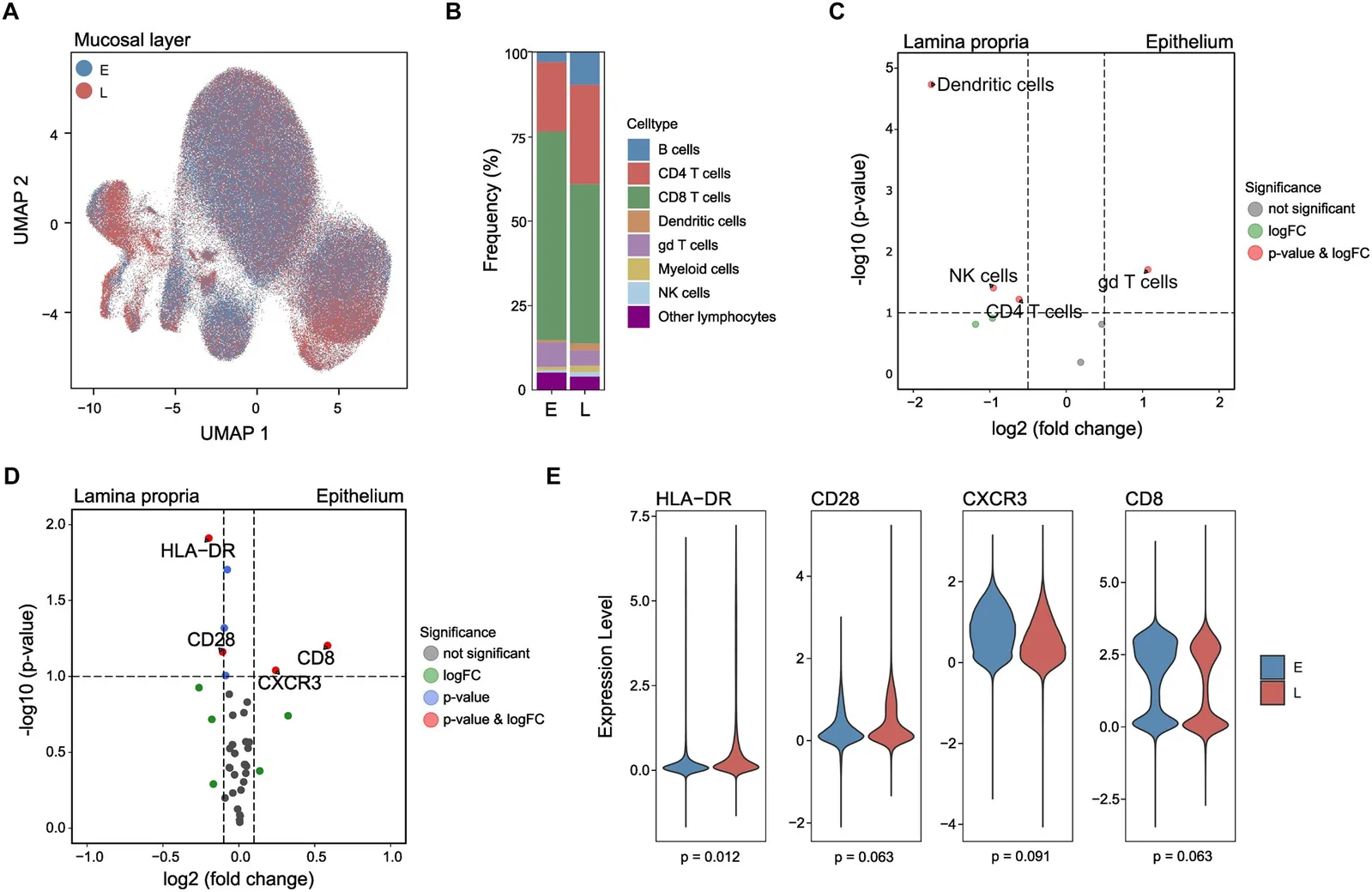
Differential abundance and differential expression analysis of immune cells in intestinal mucosal layers. (A) UMAP of all samples colored by mucosal anatomical layers. (B) Frequency plot of immune cell populations in epithelium (E) and lamina propria (L). (C) Volcano plot of differential abundance analysis comparing immune cell populations in epithelium to lamina propria (FDR < 0.1, logFC > 0.5). (D) Volcano plot of differential expression analysis using median protein signal intensity per sample comparing immune cells of epithelium to lamina propria (FDR < 0.1, logFC > 0.1). (E) Violin plots of differential protein expression across mucosal layers (*n* = 14).

To investigate differences in (surface) protein expression in cytometry data, a DE analysis can be conducted on a single-cell level using Seurat’s ‘FindMarkers’ function; however, using the median protein expression per sample (pseudobulk) provides a more conventional approach to detect differentially expressed markers in cytometry. To this end, we used the limma R package (Ritchie *et al*. 2015) to compare the median protein signal intensity per sample across conditions (DE_pseudobulk()). The function computes a linear model followed by an empirical Bayes statistic for DE (eBayes). HLA-DR and CD28 were enriched on immune cells in lamina propria samples, whereas CXCR3 and CD8 were enriched on epithelial immune cells (Fig. 5D,E). HLA-DR was present on B cells (Fig. 4D), which are known to be enriched in lymphoid follicles in the lamina propria compared to epithelial layer in intestines (Mowat and Agace 2014), whereas CXCR3 was high on CD8 T cells (Fig. 4D) and enriched in epithelial immune cells (Fig. 5D,E). In sum, the differential protein expression results are in line with the differential population abundance analysis and previous studies (Mowat and Agace 2014, Sagebiel *et al*. 2019, Schreurs *et al*. 2019). These findings highlight that the Seumetry toolkit offers a user-friendly implementation of a more conventional differential population abundance and differential protein expression analysis.

### 3.6 Workflow validation on public data

To further demonstrate the fidelity and straightforward usability of Seumetry and to demonstrate the compatibility with mass cytometry data, we analyzed a publicly available dataset previously used to evaluate the cytometry analysis packages CyTOF workflow (Nowicka *et al*. 2017) and CITRUS (Bruggner *et al*. 2014). This mass cytometry dataset contains peripheral blood mononuclear cells (PBMCs) from two treatment conditions (control and BCR/FcR-XL-treated) stained for 10 surface and 14 intracellular antigens (Bodenmiller *et al*. 2012, Bruggner *et al*. 2014, Nowicka *et al*. 2017). To explore the variance across samples in the public dataset, a PCA computed with Seumetry revealed a segregation of samples based on treatment conditions (Fig. 6A), consistent with published results (Nowicka *et al*. 2017). Seumetry clustering analysis using Seurat functions yielded 32 clusters (Fig. 6B), which were manually merged based on the expression of the immune cell lineage markers (Fig. 6C). The resulting merged clusters exhibited comparable marker expression (Fig. 6D) and frequencies of immune cell populations per condition (Fig. 6E). To assess clustering accuracy beyond this qualitative comparison, we benchmarked Seumetry against expert-gated ground-truth labels for this dataset using per-cell F1 scores across a range of clustering resolutions (Fig. S4A–C, available as supplementary data at *Bioinformatics* online). At a clustering resolution of 1.4, Seumetry recovered all eight expert-gated populations with a mean F1 score of 0.77, with the best resolved populations, such as CD4 T-cells, exceeding an F1 of 0.9 (Fig. S4A and C, available as supplementary data at *Bioinformatics* online). Lower resolutions missed some populations, while resolutions beyond 1.4 offered little further gain in F1 or population recovery but continued to reduce clustering stability (Adjusted Rand Index) (Fig. S4B,C, available as supplementary data at *Bioinformatics* online). Based on this, we recommend a degree of overclustering as the default strategy, since resulting clusters can be merged during annotation.

**Figure 6:**
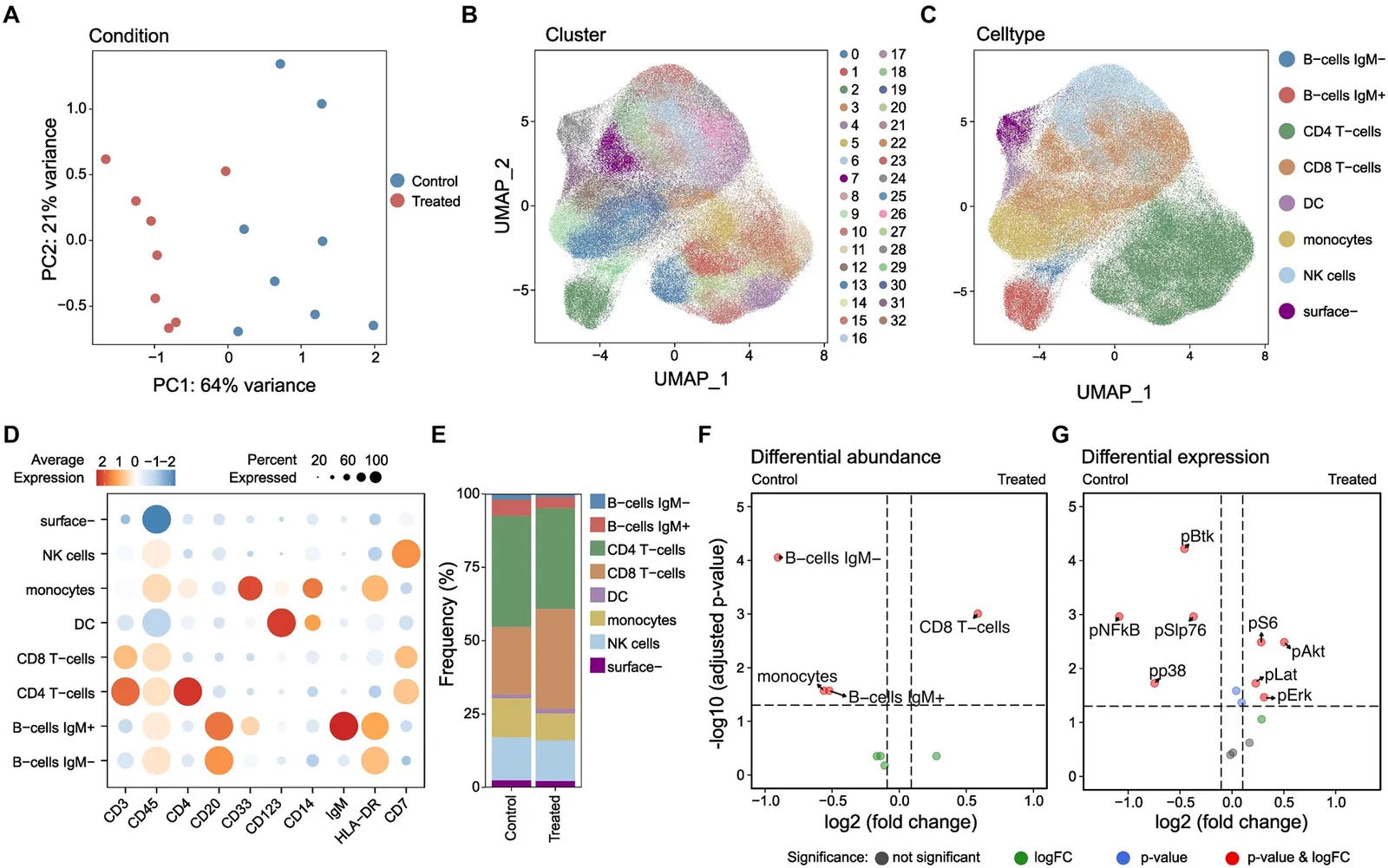
Analysis of publicly available mass cytometry data using Seumetry. (A) Principal component analysis based on median protein expression per sample colored by condition. (B, C) UMAP of all samples colored by (B) the 32 Louvain clusters and (C) merged clusters based on protein expression. (D) Dotplot of average lineage marker expression (scaled) in merged clusters. (E) Frequency plot of clusters across conditions. (F) Volcano plot of differential abundance analysis comparing stimulated to control (FDR < 0.05, logFC > 0.1). (G) Volcano plot of differential expression analysis using median protein signal intensity per sample comparing treated to control (FDR < 0.05, logFC > 0.1).

DA analysis showed that CD8 T cells increased in treated compared to control samples, while B cells decreased (Fig. 6F), matching published results (Nowicka *et al*. 2017). DE analysis identified markers such as pBtk, pAkt, pNFKB and pS6 as significantly changed in treated samples (Fig. 6G), also consistent with the published data. To formally benchmark these statistics against an established cytometry-specific method, we compared Seumetry’s DA and expression results to diffcyt (Weber *et al*. 2019) on the same dataset. Results were highly concordant: 100% agreement in significance calls, with adjusted *P*-values correlating to DA (*r* = 1.000) and DE (*r* = 0.984) (Fig. S4D,E, available as supplementary data at *Bioinformatics* online), attributed to a similar implementation of DA and DE algorithms, edgeR and limma.

To test whether Seumetry performs equally well on a more complex dataset, we applied it to a second, independent expert-gated mass cytometry dataset with 32 markers (Levine *et al*. 2015). The original ground truth annotation for this dataset includes three distinct CD34+ progenitor subpopulations, which Seumetry’s clustering was unable to resolve as separate clusters. We therefore collapsed these into a single “CD34+ cells” category for benchmarking, yielding the 12 populations reported here. This reflects a limitation of the unbiased, graph-based clustering approach: very small populations, or populations distinguished only by minor shifts in marker intensity or complex hierarchical gating strategies, may not be identified in comparison to manual gating. With donor integration, Seumetry reached a mean F1 score of 0.91 across these 12 populations, recovering more populations as clustering resolution increased. A similar stability vs. accuracy was seen as in the first dataset (Fig. S4F–H, available as supplementary data at *Bioinformatics* online), underscoring the use of the overclustering approach. Integration improved clustering accuracy over processing without donor correction (mean F1 = 0.91 vs. 0.86; Fig. S4I, available as supplementary data at *Bioinformatics* online), providing direct evidence that batch effect correction can measurably improve population recovery and annotation accuracy. All populations were clearly separated in the resulting UMAP (Fig. S4J, available as supplementary data at *Bioinformatics* online).

To empirically assess scalability of Seumetry, we benchmarked runtime, peak memory, and object size of Seumetry and Seurat functions using a high-dimensional public dataset (Samusik *et al*. 2016) with increasing numbers of markers and cells (Fig. S5, available as supplementary data at *Bioinformatics* online). Runtime and memory both scaled roughly linearly with dataset size. The full pipeline processed 5 00 000 cells across 39 markers in about 10 minutes using under 8 GB of peak memory, which is within reach of standard computing hardware.

In conclusion, Seumetry recapitulates known differences in immune cell populations from mucosal tissues, bone marrow and peripheral blood, and per-cell benchmarking against expert-gated ground truth shows its clustering and differential analysis results closely match those of established methods.

### 3.7 Concluding remarks

Here, we introduce Seumetry, a versatile R toolkit for comprehensive analysis of high-dimensional cytometry data. Seumetry comprises all necessary tools to analyze flow and mass cytometry data: (1) preprocessing (compensation, transformation), (2) quality control (outlier and antibody aggregate detection), (3) cytometry-specific visualizations, (4) DA and DE analysis. Furthermore, Seumetry offers advanced high-dimensional analysis methods such as dimensionality reduction, clustering, and compatibility with third-party scRNAseq tools through seamless integration into the Seurat framework. In comparison with existing cytometry analysis tools (Table 1), Seumetry offers advantages such as its modular workflow by direct integration into Seurat and novel preprocessing algorithms (aggregate detection).

The rapid development of cytometry technologies allows us to quantify more protein markers. With the increasing complexity of cytometry data, traditional 2-dimensional manual gating methods are becoming less suited, as they are highly time-consuming, and due to the increased likelihood of missing cell populations. The challenges of manual gating can be addressed using more automated approaches, which Seumetry provides. Seumetry does not eliminate the initial cleanup gating (removal of debris, doublets, and selection of viable, lineage-positive cells), which remains a manual step upstream of any Seumetry analysis. Automated analysis methods could facilitate high-dimensional cytometry data analysis and accelerate basic and translational research. As immunological analyses for clinical purposes are steadily increasing due to the enhanced use of biologicals and small molecules targeting immune pathways, standardized approaches such as Seumetry can aid robust and efficient immunological analyses for diagnosis and treatment monitoring.

In the future, the number of parameters will further increase, which will exacerbate the challenges facing manual gating. In contrast, increasing data complexity can improve the unbiased analysis of cytometry data using Seumetry, since more information will be available to classify cell types and cell phenotypes. Seumetry is scalable, as demonstrated by our benchmarking (Fig. S5, available as supplementary data at *Bioinformatics* online), and its interoperability with efficient scRNAseq toolkits ensures it will continue to benefit from ongoing improvements in the scRNAseq analysis ecosystem.

One of the biggest challenges with cytometry data is the presence of noise, due to cell debris/clumps, apoptotic cells, or other artifacts such as antibody aggregates (Finak *et al*. 2012, Rasmussen *et al*. 2021, Emmaneel *et al*. 2022). Preprocessing in Seumetry improves data quality by detecting noisy events in an unbiased manner. However, data quality is highly dependent on the source of cells/tissue and the processing in the laboratory. To alleviate these issues, we included multiple parameters that researchers can use to tailor the algorithm to each dataset, which should facilitate data-specific detection of noisy events based on scientific requirements.

In summary, Seumetry offers a versatile and scalable R framework for the unbiased and comprehensive computational analysis of high-dimensional flow and mass cytometry data, which will be valuable for extracting meaningful biological information and facilitating the development of novel methods for cytometry data analysis.

## Supplementary Material

btag730_Supplementary_Data
