# Supplementary material for: Seumetry: a versatile and comprehensive R toolkit to accelerate high-dimensional flow and mass cytometry data analysis
Source: Bioinformatics. 2026 Oct 13;42(10):btag730. doi: 10.1093/bioinformatics/btag730 (PMC13675103; doi:10.1093/bioinformatics/btag730)
Supplement: Supplementary file 1 [file btag730_supplementary_data.zip › supplementary_figures.pdf]

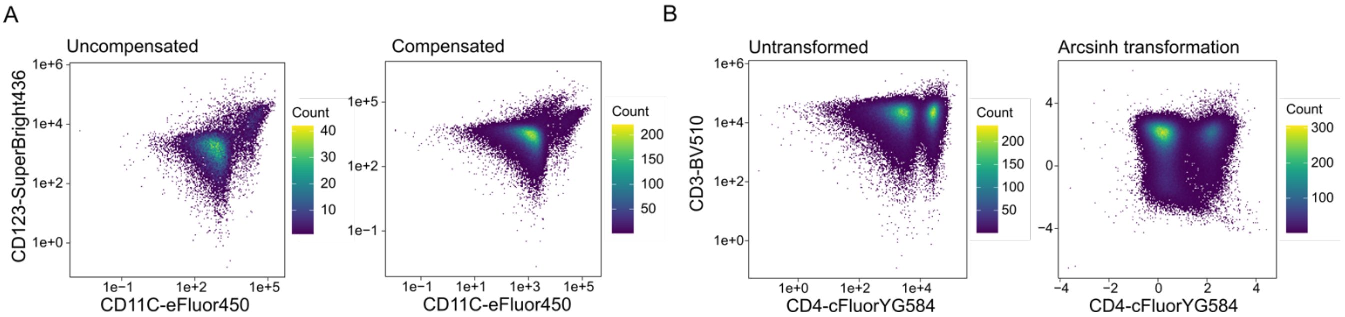

Figure S1. Compensation and transformation. (A) Representative two-dimensional density plots before and after compensation using CD11C and CD123 as exemplary markers (n=14). (B) Representative two-dimensional density plots of untransformed and inverse hyperbolic sine function (arcsinh) transformed data using CD3 and CD4 as exemplary markers (n=14).

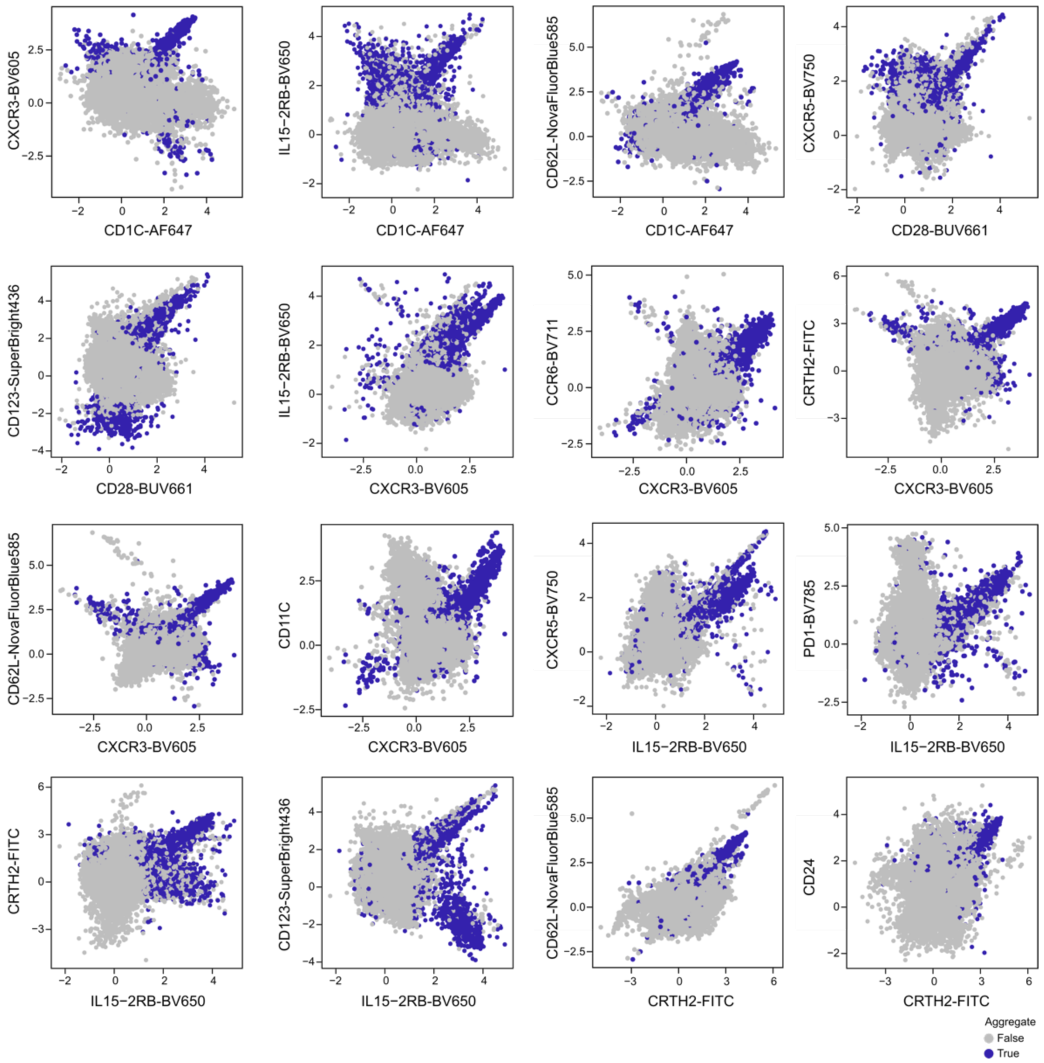

Figure S2. Antibody aggregate events across all channels. Antibody aggregates are detected using our novel algorithm on all channel combinations with a high likelihood of antibody aggregates, which are identified by Pearson correlation. Scatterplots depict identified antibody aggregates across all channels (blue) and non-aggregates (gray).

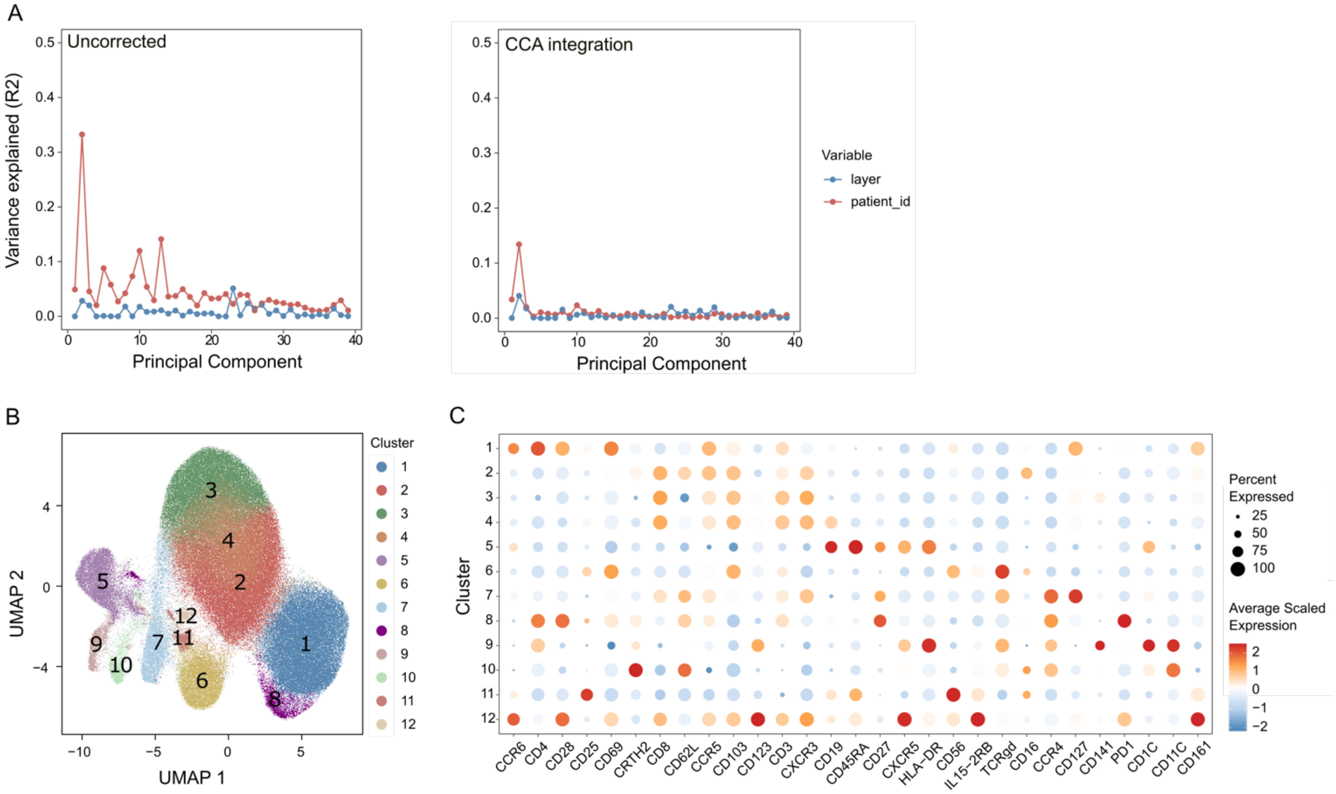

Figure S3. Merging and annotation of Louvain clusters identifying intestinal immune cell populations. (A) Linegraphs visualizing the explained variance (R squared of linear regression) of patient_id (donor) and mucosal layer variables associated with each principal component. (B) UMAP of all samples colored by Louvain clusters. (C) Dotplot of average expression (scaled) of top 5 cluster markers per cluster identified using Seurat's “FindAllMarkers” function (n=14).

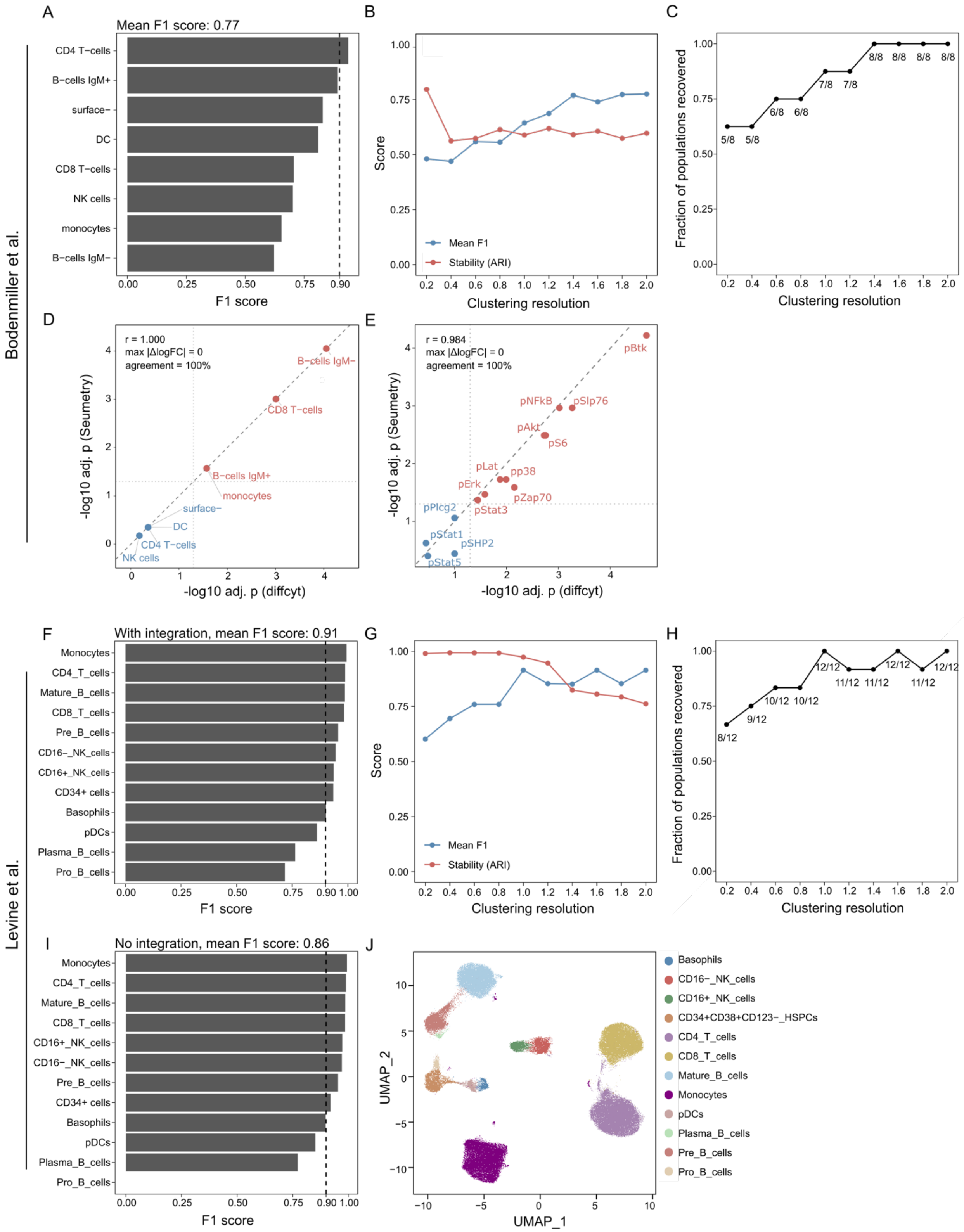

Figure S4. Benchmarking of Seumetry using publicly available cytometry datasets. (A-E) Bodenmiller et al. mass cytometry dataset (Bodenmiller *et al.* 2012). (A) F1 score per cell population at clustering resolution of 1.4 based on ground truth expert-gated populations (mean F1 = 0.77). (B) Mean F1 score and clustering stability (Adjusted Rand Index, ARI) across clustering resolutions. (C) Fraction of populations recovered across clustering resolutions. (D) Differential abundance -log10 adjusted p-values, Seumetry vs. diffcyt (r = 1, agreement = 100%). (E) Differential expression -log10 adjusted p-values for markers, Seumetry vs. diffcyt (r = 0.984, agreement = 100%). (F-J) Levine et al. mass cytometry dataset (Levine *et al.* 2015). (F) F1 score per cell population with donor integration based on ground truth expert-gated populations (mean F1 = 0.91). (G) Mean F1 score and clustering stability (ARI) across clustering resolutions. (H) Fraction of populations recovered across clustering resolutions. (I) F1 score per cell population without donor integration (mean F1 = 0.86). (J) UMAP of all cells colored by Seumetry annotated cell population.

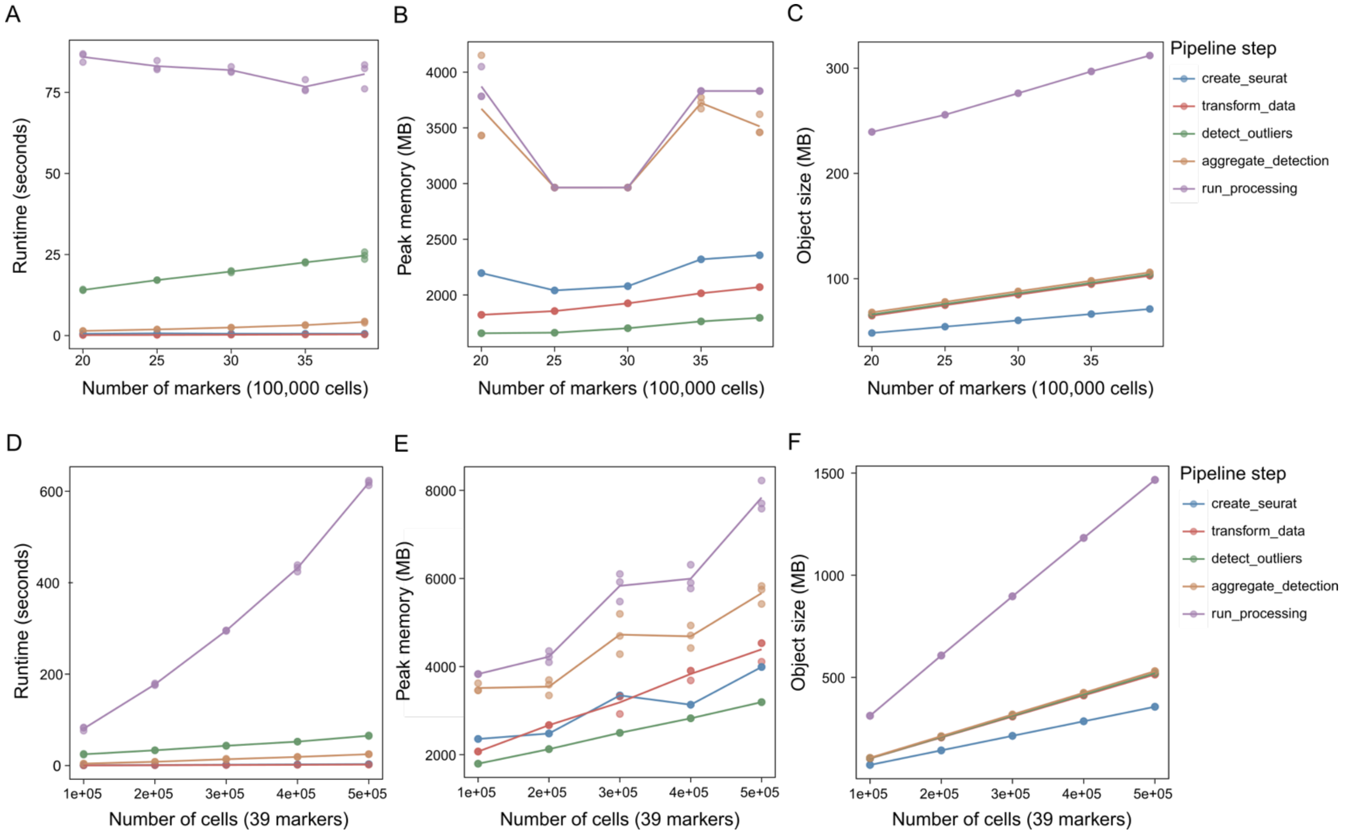

Figure S5. Scalability benchmarking of the Seumetry pipeline. A public high-dimensional mass cytometry dataset was used to benchmark Seumetry (Samusik *et al.* 2016). (A–C) runtime, peak memory, and Seurat object size as a function of the number of markers (20–39) at 100,000 cells. (D–F) Runtime, peak memory, and object size as a function of the number of cells (100,000–500,000) at 39 markers. Individual Seumetry steps (create_seurat, transform_data, detect_outliers, aggregate_detection) and the Seurat processing including PCA, UMAP, and clustering (run_processing) are shown separately.
